# Eye-Tracker Study of Influence of Affective Disruptive Content on User’s Visual Attention and Emotional State

**DOI:** 10.3390/s22020547

**Published:** 2022-01-11

**Authors:** Anna Lewandowska, Izabela Rejer, Kamil Bortko, Jarosław Jankowski

**Affiliations:** Computer Science and Information Technology, West Pomeranian University of Technology, 70-310 Szczecin, Poland; irejer@zut.edu.pl (I.R.); kbortko@zut.edu.pl (K.B.); jjankowski@zut.edu.pl (J.J.)

**Keywords:** affective pictures, emotions, eye-tracking, face emotion recognition, advertisements

## Abstract

When reading interesting content or searching for information on a website, the appearance of a pop-up advertisement in the middle of the screen is perceived as irritating by a recipient. Interrupted cognitive processes are considered unwanted by the user but desired by advertising providers. Diverting visual attention away from the main content is intended to focus the user on the appeared disruptive content. Is the attempt to reach the user by any means justified? In this study, we examined the impact of pop-up emotional content on user reactions. For this purpose, a cognitive experiment was designed where a text-reading task was interrupted by two types of affective pictures: positive and negative ones. To measure the changes in user reactions, an eye-tracker (for analysis of eye movements and changes in gaze points) and an iMotion Platform (for analysis of face muscles’ movements) were used. The results confirm the impact of the type of emotional content on users’ reactions during cognitive process interruptions and indicate that the negative impact of cognitive process interruptions on the user can be reduced. The negative content evoked lower cognitive load, narrower visual attention, and lower irritation compared to positive content. These results offer insight on how to provide more efficient Internet advertising.

## 1. Introduction

Intrusive web advertising, such as animated, pop-up, layer ads, and other informational content appearing in any part of the screen, drag the users’ attention away from their primary task: reading or navigating through the main content of web pages. This approach is a standard procedure used by advertisement designers who use their marketing activities to ensure advertisements are delivered and noticed. Therefore, increasing the effectiveness of ads delivery is often accompanied by an increase in intrusiveness [1,2,3]. However, as was observed in early studies related to TV marketing [4], the increased usage of invasive forms of advertising leads to ad avoidance. This pattern strengthens for various forms of interactive media such as pop-up ads [1], video content [5], and within social media [6].

The distractions introduced by online ads are being perceived as annoying by an increasing number of users, which has created a negative attitude toward the ads. Irritation is associated with most forms of intensive advertising, especially with forced exposure such as pop-up, layer [1], and pre-roll video ads [2]. Therefore, advertising companies are facing the dilemma of how to draw users’ attention to the disruptive content without evoking their negative attitude. To limit the negative impact of ads on consumers, various techniques can be used such as increasing ad relevance [7,8], including entertaining content [9], increasing privacy protection [10], using gamification [11], or increasing user control [12]. Entirely eliminating the negative feelings evoked by interruptions may be challenging, but it is at least possible to lower their intensity by providing the content of emotional load or highly engaging content, thereby attracting user attention.

With the above assumptions, adding emotional appeal to video pre-roll ads has received special focus. Li et al. reported the results of an experiment where a level of intrusiveness was measured with a scale [3], showing that ads with positive emotional load appeal are considered less intrusive and generate more positive attitude toward the advertising content and the brand. However, the results of extended study where more emotional states were considered [13] showed that the usage of basic emotions such as disgust, happiness, sadness, surprise, and suspense within pre-roll video ads would not help to stop or reduce irritation, regardless of their valence. The study confirmed that the basic emotions significantly increase the skipping of video ads, but complex affective responses such as humor, fun, and warmth decrease skipping and can help to increase ad effectiveness. Another study verified the possibility of decreasing perceived intrusiveness by adding emotional appeal to pre-roll ads displayed with video content, where higher positive emotional appeal led to lower perceived intrusiveness [14].

Emotional load is also known as a factor modifying visual attention. The influence of affective pictures on attention is reported in [15,16,17]. Most of these effects were originally reported for negative stimuli (snakes, fearful, or angry faces). The results showed that stimuli with negative valence are detected rapidly and shift visual attention to their location [15,18,19]. Conversely, positive affect is thought to promote the exploration of new information, in contrast to negative emotions, which primarily act to focus (narrow) attention and cognition. In support, authors [20,21] demonstrated that positive mood states widen the range of attention to visual and conceptual space. Such results suggest that positive valence, at least in terms of mood, can serve to broaden one’s range of attention, unlike the effects of negative valence, which narrow the distribution or scope of one’s attentional field of view. The effects of wide and narrow attention are called ambient and focal attention, respectively, in the eye-tracking literature. Ambient attention is typically characterized by relatively short fixations followed by long saccades. Conversely, focal attention is described by long fixations followed by short saccades [22,23].

A separate study investigating how the arousal level of a stimulus influences visual attention assumed that highly arousing stimuli capture attention, regardless of their emotional valence [24]. Thus, according to arousal theories of attention, it is arousal rather than valence that influences the amount of attention that is voluntarily or involuntarily focused on stimuli.

The studies presented above analyzed the influence of the emotional content on users’ visual attention or their emotional response. In this study, our aim was to study both factors simultaneously and to determine whether such an approach allows for a more compact and unified answer to the question on how to prepare the marketing content to reduce user irritation and limit ad avoidance. Hence, the aim of this study was to measure the impact of affective disruptive content and its emotional type on changes in user visual attention, cognitive load, and emotional states.

**Figure 1:**
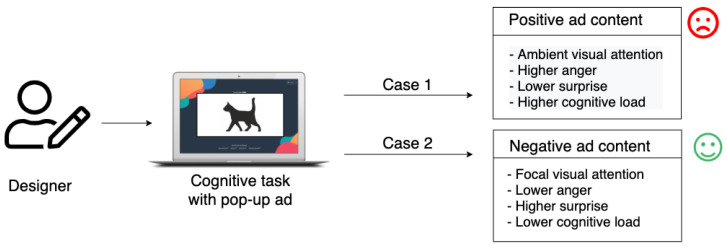
Problem definition.

To achieve our aim, we designed a cognitive experiment. During the experiment, a subject’s task was to read and understand the text displayed on the screen. The text-reading task was suddenly interrupted by affective pictures (taken from the International Affective Picture System (IAPS) [25]) appearing in the center of the screen (Figure 1). Although different kinds of emotions can be evoked by those pictures, we based our study on the most general classification of emotion that divides the whole spectrum of emotions into two classes: positive and negative. Thus, positive and negative pictures were used without adding subclasses or levels of intensity to create sharp differences between the used stimuli. During the experiment, we collected two types of data: eye movements and gaze points identification (acquired by an eye-tracker) and facial muscle micro-movements (recorded with an iMotion platform). Whereas the eye movements were recorded to detect changes in visual attention and the cognitive load, the patterns of facial muscle movements were acquired to measure the level and type of emotions felt by users. Notably, our goal was not to recognize the primary emotions, such as happiness, anger, sadness, etc.

The results of our experiment confirm that the choice of emotional load of the disruptive content is an important factor when designing ads. The correct type of emotional content might reduce the negative impact of interrupting the user’s ongoing cognitive process. As we found, the more negative the content, the lower the cognitive load, and the narrower the visual attention, and the lower the irritation.

The paper is structured as follows. Section 1 presents the motivation behind the study. All the materials and methods used in the research can be found in Section 2. Section 3 discusses the results of our experiment, and Section 4 concludes the paper.

## 2. Materials and Methods

### 2.1. Experiment Setup

**Figure 2:**
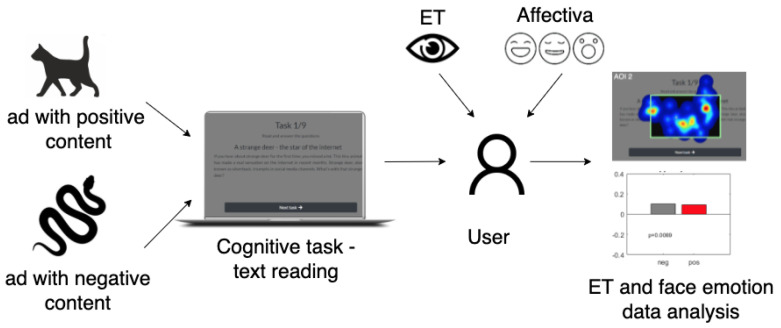
Experiment overview.

The goal of the experiment was to measure the impact of affective disruptive content and its emotional type on user visual attention, cognitive load, and emotional states change (Figure 2). The experiment was conducted with 33 respondents who declared normal or corrected-to-normal vision. All analyzed data were fully anonymized. Before the experiment, the participants provided informed written consent to have data from the perceptual experiment used in research (according the Bioethics Committee Agreement no KB-0012/24/2020).

The experiment was performed using an NEC monitor with a native resolution of 1680 × 1050 pixels. The monitor display was calibrated to the sRGB color space using a Minolta CS-200 colorimeter and a Specbos 1201 spectroradiometer. During the experiment, the gaze points and eye movements were recorded with a Tobii Pro X3 120 Hz eye-tracker, and the movements of the user’s face muscles were recorded with an iMotions platform. The recording device (HP webcam HD 3300 720P) was attached to the upper part of the stimulus screen.

**Figure 3:**
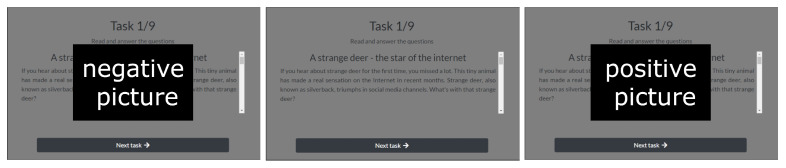
The course of the experiment and the experimental setup. (**Left**): The negative stimuli. (**Middle**): The cognitive task. (**Right**): The positive stimuli. As according to the signed agreement, pictures from the IAPS database cannot be shared outside of research, so they are presented with their names and characteristics in Table 1.

The main respondent task was to read a set of 10 different texts; each text was followed by a short questionnaire testing a text understanding. The reading process was interrupted by visual stimuli with a positive or negative emotional load. The examples of negative and positive stimuli displayed during the experiment, as well as the text reading task, are presented in Figure 3.

The two-level randomization procedure was applied in the process of selecting the stimuli for each respondent. First, the order of the condition (negative/positive) was randomly chosen; second, the pictures in one emotional group were randomly arranged. Such an experimental plan enabled us to find out whether there are statistically significant differences between the two experimental conditions, that is, between positive and negative stimuli.

The disrupting stimuli were presented for seven seconds with random intervals (13 and 26 s) between each two of them. In total, 30 stimuli were displayed for each user. After displaying the whole set of 30 stimuli (15 negative and 15 positive), the remaining part of the reading task was free from interruptions. The average experiment time was about 15–20 min.

The affective pictures used in the experiment were obtained from the IAPS database [25], which was used for the assessment of human emotions and the assignment of experimental tasks [26]. The IAPS database consists of approximately 900 visual stimuli (photographs) provided in three-dimensional values (arousal, dominance, and valence) of subjects’ emotional responses, which are assessed through SAM for each photograph [27]. For the experiment, a total of 30 IAPS photographs (15 photographs for each group of positive and negative) were selected based on the following valence values in each group: negative, 4 points or less; positive, 6.3 points or more. According to the signed agreement, pictures from the IAPS database cannot be shared outside of research; therefore, we present them with their names and characteristics in Table 1. The visualization of the experiment in Figure 3 is only for illustrative purposes.

**Table 1:** The valence, arousal, and dominance data of the IAPS photographs used in the experiment. The characteristics of the pictures were obtained from [25,27].

Type of Emotion	Description	Slide No.	Valence Mean (SD)	Arousal Mean (SD)	Dominance Mean (SD)
Negative	Snake	1050	3.46 (2.15)	6.87 (1.68)	3.08 (1.93)
	Snake	1120	4.73 (1.75)	6.60 (1.38)	4.67 (1.10)
	Pit Bull	1300	4.06 (1.54)	6.90 (1.59)	3.67 (1.88)
	Shark	1930	4.12 (1.92)	5.98 (2.24)	3.56 (2.43)
	AngryFace	2120	3.65 (2.05)	4.93 (2.46)	5.30 (2.22)
	SadChild	2800	2.31 (1.36)	4.94 (1.97)	4.00 (2.41)
	Tornado	5970	4.31 (1.64)	4.65 (2.61)	3.79 (2.54)
	ElectricChair	6020	4.10 (2.15)	5.23 (2.21)	4.65 (2.59)
	AimedGun	6250	2.98 (1.97)	6.35 (2.74)	2.70 (1.91)
	AimedGun	6260	2.53 (1.63)	7.10 (1.90)	2.92 (2.25)
	Knife	6300	3.30 (1.67)	6.37 (1.73)	3.41 (2.08)
	BarbedWire	9010	3.68 (1.57)	4.32 (1.89)	4.02 (2.04)
	Seal	9180	2.76 (1.36)	5.07 (2.26)	4.44 (2.40)
	Skulls	9440	4.42 (1.82)	4.71 (1.97)	5.27 (2.47)
	DuckInOil	9560	2.07 (1.89)	5.46 (2.60)	3.41 (1.85)
Positive	Dog	1500	7.72 (1.70)	4.15 (2.46)	6.90 (1.71)
	Rabbit	1610	8.39 (0.91)	3.33 (2.36)	6.56 (2.15)
	Girl	2304	7.70 (1.11)	3.91 (2.28)	6.25 (1.90)
	ElderlyWoman	2510	7.13 (1.90)	3.88 (2.17)	5.44 (2.12)
	Boy/ice-cream	2650	6.82 (1.91)	4.25 (2.48)	6.02 (1.75)
	Boy	5030	7.71 (1.26)	4.31 (2.37)	5.47 (1.94)
	Galaxy	5300	6.96 (1.86)	4.09 (2.64)	4.56 (2.75)
	Fireworks	5480	7.69 (1.45)	5.41 (2.34)	5.71 (1.89)
	IceCream	7330	7.96 (1.49)	5.54 (2.53)	6.46 (2.58)
	Pancakes	7470	7.18 (1.70)	4.72 (2.20)	5.42 (1.69)
	Athlete	8120	7.15 (1.40)	4.60 (2.16)	6.29 (1.79)
	WaterSkier	8200	7.86 (1.12)	6.37 (1.94)	6.13 (1.67)
	Runner	8465	6.61 (1.37)	4.93 (2.24)	6.10 (1.54)
	Money	8502	7.65 (1.78)	6.00 (2.55)	6.29 (2.71)
	SportCar	8531	7.11 (1.65)	5.25 (2.28)	6.55 (1.92)

### 2.2. Eye-Tracking Metrics

Eye movement characteristics provide indirect access to cognitive processes, e.g., decision making [28], attention [29], and memory [30]. Particularly, ocular events, e.g., saccades, blinks, fixations, and pupillary responses, involve different neural circuitries in connection with visuomotor information processing [31]. Fixation duration has often been investigated in the context of visual attention mode, e.g., ambient mode, which is characterized by short fixations and long saccades during early scene inspection, and focal mode, which is characterized by longer fixations, which is associated with more detailed object feature processing during later inspection phases [32].

A number of studies [33,34,35] have suggested that saccade velocity (SCV), saccade amplitude (SCA), saccade duration (SCD), fixation duration (FD), blink duration (BD), blink frequency (BF), and pupil dilation range (PDR) may be sensitive to mental load variation and fatigue. According to [36], saccade is a relevant ocular event for studying fatigue and the following decrease in user attention. The velocity of saccades is an especially good indicator of stress, mental overload, irritation states, and lowering attention oculometrics. Saccade velocity is the average saccade speed in degrees per second. A higher saccade velocity indicates higher stress and task complexity and lower concentration while performing a task. The higher the cognitive load, the shorter the saccades, and the higher the saccade velocity [37].

In our research, to measure visual attention change, when interruption from a cognitive task occurred, we chose two metrics: fixation duration, in the form of fixation duration in the area of interest (AOI) of a given stimuli compared to the fixation duration in the experiment window for the total time the stimuli was displayed (fixation duration percent (FDP); and the saccade velocity (SCV). Notably, according to [1], information or advertisement that is deemed important, interesting, or intriguing rewards the viewer, who is thereby less likely to feel irritated by the interruption. Therefore, in our research, we wanted to check if the value that viewers receive from advertising could be increased by considering the emotional message contained in the ad.

### 2.3. Eye-Tracker Data Preprocessing

The eye-tracker enabled us to register the gaze points and eye movements and thus to isolate the elements that attracted the user’s attention at a given moment. To analyze the eye-tracker data, the metrics were computed per every picture that interrupted the text reading process for every observer. However, before the statistical differences between data were computed, the saccadic velocities were standardized with Equation (1).

(1) $$z_{i,j}=\frac{d_{i,j}-\bar{d_{i}}}{\sigma_{i}},$$

where *i* is the observer number (i∈(1,…,n)), *j* is the picture number (j∈(1,…,m)), $d_{i,j}$ is the value of saccadic velocity, $\bar{d_{i}}$ is the mean value of saccadic velocity, and $\sigma_{i}$ is the standard deviation of saccadic velocity.

The fixation duration percent ($FDP_{k}$) is described according to Equation (2), so the values did not require standardization.

(2) $$FDP=\frac{FDAOI_{k}}{FDS_{k}},$$

where $FDAOI_{k}$ is the fixation duration in the area of interest $AOI_{k}$ defined for stimuli *k*, and $FDS_{k}$ is the fixation duration in the experiment’s window during the time when the stimuli was displayed (where stimuli *k* was presented).

Next, as the observers may have received implausible impression scores because they misunderstood the experiment instruction or did not engage in the task and provided random answers, a screening procedure was employed. For this, we applied the standard approach described in [38], Annex 2.3.1, that provides a numerical screening procedure. The procedure involves counting the number of trials in which an observer’s result lies outside the ±2 standard deviation range and rejecting those observers for whom (a) more than 5% of the trials are outside that range and (b) the trials outside that range are evenly distributed so that the absolute difference between the counts of trials exceeding the lower and upper bounds of that range is not more than 30%. We applied this procedure to our data but did not find any participants that needed to be removed.

### 2.4. Analysis of Emotions

The complexity of emotional response to presented stimuli allows measurement of the user’s emotional state from many different perspectives. Among the approaches used for assessing human emotional responses, two main methods can be distinguished. The first is based on analysis of reactions hidden inside the biosignals measured from the human body by using methods such as electroencephalography (EEG) [39], electrocardiography (ECG) [40], electromyography (EMG) [41], galvanic skin response (GSR) [40,42,43], and eye-tracking (ET) [44]. The second is focused on analyzing more external responses, such as body gestures [45], speech [46], or facial expressions [47,48,49,50]. Although all these approaches have been extensively studied, in the domain of emotional expression, the movements of facial muscles are regarded as the central source of information [51,52].

Facial expression analysis is usually based on the facial actions coding system (FACS) published in 1978 by Ekman and Friesen [53]. The system was first created in 1970 by Hjortsjö [54]; then, it was enhanced and published by Ekman in 1978 and once again updated in 2002 [55]. FACS describes a set of facial muscle movements coded in the form of action units (AUs). A single AU corresponds to a contraction or relaxation of one or more muscles. The FACS defines 44 AUs [51], such as AU7, lid tightener; AU14, dimpler; AU24, lip pressor; etc. The intensities of AU movements are measured on a discrete scale, ranging from A (trace) to E (maximum). The FACS alone does not provide any direct emotion descriptors [47]. The classification of emotions is based on patterns of AU activations that are described in related sources [56]. The sets of patterns corresponding to 6 basic emotions (anger, disgust, fear, happiness, sadness, and surprise) are additionally listed in [47].

Although the manual assignment of scores to different AUs to recognize underlying emotional expressions is possible for static pictures or short video clips, the emotional tagging of longer video material is ultimately a tedious task. Therefore, automated recognition systems are usually used to deal with the task. Apart from different algorithms and approaches proposed by scientists from leading research centers [57,58,59], fully commercial systems such as Azure Face API, Face++, Noldus FaceReader, or the iMotion module for Facial Expression Analysis [60,61,62] are available on the market. In our work, we used the latter of the mentioned systems, the iMotion module, which uses the Affectiva algorithm [63] to detect AU movements and the underlying emotions.

### 2.5. Emotional Data Preprocessing

Although the Facial Expression Analysis module provides both the raw information about the activation of single action units and their translation to core emotions (joy, anger, fear, disgust, contempt, sadness, and surprise) together with some additional indexes, only nine features were stored for offline analysis. Seven of them represented the core emotions mentioned above, and two represented more general mental characteristics: attention and engagement. Each of these features was provided as an intensity score measured in a normalized scale from 0 to 100.

All nine features were processed according to the same processing pipeline, which was composed of three steps. The first step was performed over the whole set of features, which was accompanied by the stimulus vector. The task of this step was to remove all empty records that were introduced during the recording process as a result of massive changes in a subject’s position that temporarily broke the contact between the camera and the subject. Usually, empty records appeared when the picture box was removed from the screen at the beginning of the next text-reading period. The average number of empty records was about 10% of the total.

All the next steps from the processing pipeline were performed individually on each feature. The task of step 2 was to remove the influence of the outlier data. This step was necessary because each sudden movement of the subject’s head introduced a large change in the feature value. To deal with the outlier problem, we calculated the 5th and 95th percentiles over the feature time series and replaced all the feature values falling under the 5th percentile or exceeding the 95th percentile with the value of the 5th or 95th percentile, respectively. In step 3, we applied 250 ms nonoverlapping windows on the feature time series. For each window, a corresponding label (–1, text-reading period; 1, negative picture presentation; 3, positive picture presentation) was assigned. Next, each window was described by its means value.

At the end of the processing pipeline, about 260 samples characterizing negative and 260 samples characterizing positive picture periods were obtained for each subject (10 pictures × (4 windows per second × 7 s of 1 picture presentation − 2 possible borders periods)). Regarding the text-reading periods, the number of samples characterizing those periods varied among subjects. This number of samples was much higher since (i) the text was presented before each picture and (ii) the text presentation time was longer. On average, about 3560 samples characterizing text periods (20 text periods × (4 windows per second × 45 s of 1 text presentation − 2 possible border periods)) were obtained for each subject. Hence, the total size of the feature matrix of one subject was about 9 features × 4080 samples. To ensure the comparability of the feature matrixes between subjects, each feature in the matrix was standardized to have zero mean and unit standard deviation. The feature matrices (and the stimulus vectors) calculated for all the subjects were concatenated and submitted to statistical analysis. Since the data distribution in the three analyzed groups significantly deviated from the normal distribution for most features, in all analyses, the non-parametric Kruskal–Wallis test with a *p*-value set to 0.05 was used to test the significance of between-groups differences.

## 3. Results and Discussion

The following section discusses results from the perceptual experiment with the goal to determine whether, during advertising design, both users’ visual attention and their emotional response can be considered to reduce the user irritation and limit ad avoidance.

The user’s natural reactions such as gaze point change, eye movements, and resulting oculometrics of eyesight behavior were deeply analyzed, as well the user’s natural face expression. To reliably measure the user’s natural responses, the keys were the eye-tracker and the iMotions Facial Expression Analysis Module employed during the experiment.

### 3.1. Eye Tracking

In order to determine if the negatively loaded content attracted more focal user attention and incurred lower cognitive load compared to positively loaded content, an eye-tracking signal was acquired from the experiment and analyzed with two metrics: ratio of fixation duration in the area of interest (AOI, covering the analyzed stimuli) to fixation duration on the slide of the experiment where the stimuli was displayed (FDP); and saccade velocity (SCV) when the affective picture was displayed.

**Figure 4:**
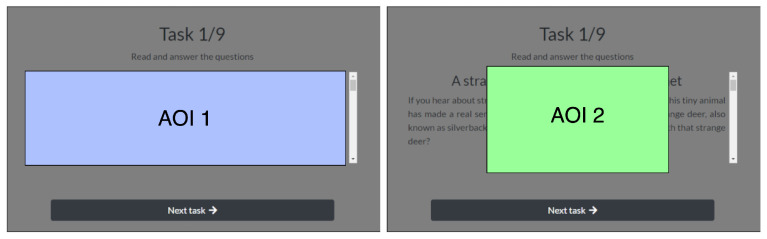
The AOIs defined for filtering the eye-tracker signal for (**left**) AOI 1, the displayed cognitive text task; and for (**right**) AOI 2, the affective picture that interrupts the cognitive process.

To analyze the eye-tracker signal in relation to the displayed stimuli (text and picture), two areas of interest were defined: AOI 1 (covering the part of the screen where only the text was displayed) and AOI 2 (covering the part of the screen where the disruptive affective pictures were suddenly displayed during the text reading). The defined AOIs for en example screen from the experiment are depicted in Figure 4.

The oculometrics used in the analysis of eye-tracking data (FDP and SCV oculometrics) are based on the main eye movements:

- Fixations are the most common feature recorded by eye trackers, providing inferences about cognitive processes or states that users are interested in probing. Fixations are the times when a user’s eyes essentially stop scanning the scene, holding the central foveal vision in place so that the visual system can obtain detailed information about what is being looked at.
- Saccades are voluntary eye movements between two fixations, and they can be visualized as scan paths. Saccadic eye movements are identified with their amplitude, duration, and velocity. The average duration of a saccade is very short, typically between 20 and 80 ms.
  The measurements of saccade velocity help observing the pattern of a scan path and exploring the cognitive effort. Saccade velocity is highly correlated to discriminatory parameters in terms of cognitive performance [64].

**Figure 5:**
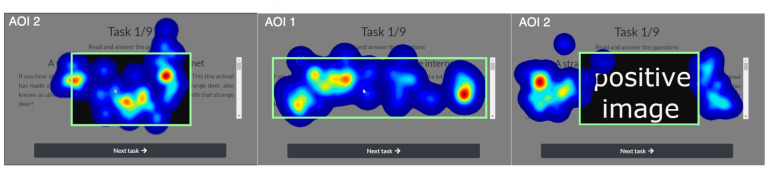
(**left**) Heat map for negative picture, (**middle**) heat map for clear text, and (**right**) heat map for positive picture. The consent to use the IAPS database [25] for scientific research is subject to restrictions prohibiting the publication of pictures in an open manner (on the Internet or directly in the publication), as we mentioned previously. Therefore, pictures of contrary emotional nature are presented only in a schematic manner.

The first oculometrics analyzed was fixation duration percent (FDP): the longer the fixation time, the more often the component attracts the user’s attention. Therefore, we analyzed the relation between fixation duration in the area of interest of a given stimuli and the fixation duration in the experiment window during the period when the stimuli was displayed (see Equation (2)). To visualize this, the heat maps for all three groups of stimuli (text, and positive and negative pictures) for the given AOIs are depicted in Figure 5.

**Figure 6:**
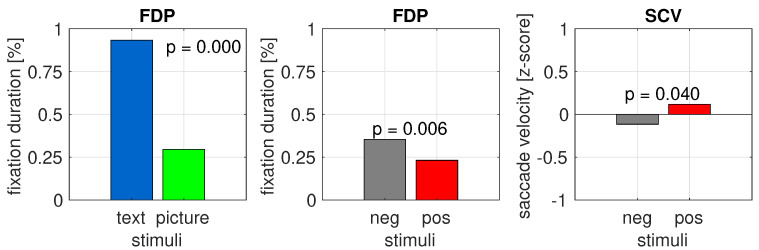
(**left**) The difference in mean fixation duration percent (FDP) between text reading (blue) in AOI 1 and disruptive pictures watching (green) in AOI 2. (**middle**) The difference in mean fixation duration percent in AOI 2 between negative pictures (gray) and positive pictures (red). (**right**) The difference in mean velocity of saccades between negative (gray) and positive (red) stimuli. The *p*-value indicates the statistical difference between compared stimuli, which is computed by the Kruskal–Wallis rest at a 0.05 significance level.

When analyzing the eyesight focus for text reading, AOI 1 was considered. For emotional pictures that interrupted the text-reading process, AOI 2 was analyzed respectively. First, we compared the text and affective pictures without division of positive and negative pictures (Figure 6 (left)). The results of the one-way two-level Kruskal–Wallis test (with 5% significance level) for fixation time spent during text reading and disruptive pictures is obvious, below $10^{\left(-17\right)}$. The results of the Kruskal–Wallis test analysis indicate a significant difference between text reading and emotional pictures. This means that the user was definitely more focused on reading the text than on the picture that interrupted this task. This situation is not surprising; however, between pictures with opposite emotional characteristics, the statistical significance appeared as well (Figure 6 (middle)). This means that the perceptions of the affective stimuli differed. The significant difference was identified between the 10 first affective pictures (p=0.006).

Despite the pictures being stimuli interrupting the user’s cognitive process, the subjects paid attention to them to different extents depending on their emotional type. Much more time was devoted to the negative than to the positive pictures. This is according to the rule of more intriguing objects attracting attention, and attention being focused on negative stimuli, which is consistent with the results previously reported [15,18,19]. The results from our experiments indicate that the negative pictures were more interesting to the users than the positive ones, and users devoted more time to them.

The second analyzed oculometrics was mean saccade velocity (SCV), which was measured when emotional stimulus was disrupted. Saccadic eye movements bring the line of sight to details of interest in the visual scene. In earlier research [33,34,35,36,37], saccade velocity has been identified as a very good indicator of fatigue, stress, mental overload, irritation states, and lowering attention oculometrics; it indicates a high degree of distraction during the gaze concentration process. In other words, SCV indicates the stability of a participant’s gaze: a more stable gaze yields a smaller saccade velocity and less chaotic eye pattern; a higher average saccade velocity indicates higher stress and lower concentration while performing the cognitive task. The higher the cognitive load, the shorter the saccades, and the higher the saccade velocity [37].

Given the above, we used the saccade velocity to prove that the emotional characteristic of disruptive stimuli (positive or negative) has an impact on user attention, especially sight stability, and on cognitive load when the cognitive task was disrupted. As the characteristics of saccades differ for text reading and looking at pictures, for the analysis of saccadic metrics, we only considered the affective stimuli. When examining the sight behavior during text reading, the saccade velocity is high, which is the nature of reading. During picture watching, the metrics should be much lower if the user is in focus.

Analyzing the results obtained from the experiment (Figure 6 (right)), we found that the standardized velocity of saccadic movements for the negative pictures was lower than for the positive pictures. The difference in mean saccades velocity between pictures of different emotional characteristics according to the one-way, two-level Kruskal–Wallis test (at a 5% significance level) was statistically significant (p=0.04). The SCV metrics showed a greater cognitive load and irritation in the case of positive pictures in comparison to the negative ones. This finding is most likely related to the fact that the pictures were so intriguing and surprising that they eliminated the irritation resulting from the interruption of the cognitive process in comparison to the positive pictures. This could also have resulted from the mechanism where negative feeling can be reduced by other negative impacts.

### 3.2. Emotions Recognition

**Figure 7:**
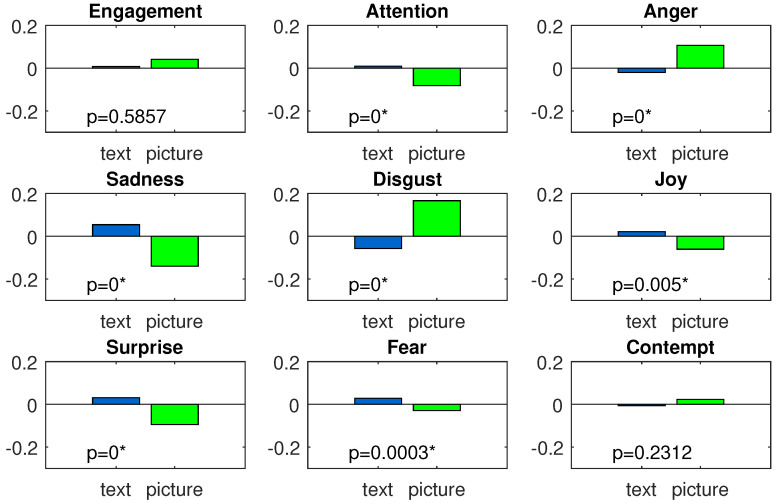
Emotional features: total means for text and picture conditions; the significant differences in both conditions (at 0.05 level) are indicated with an asterisk.

To identify which emotions were evoked when the subject’s cognitive task (text-reading task) was interrupted with emotional pictures, we used a one-way statistic test. In this test, we were not interested in picture valence: we only wanted to check whether the emotional features significantly differed between the two conditions: the text-reading condition and the picture presentation condition. To prepare data for this analysis, the samples labeled as negative and positive were joined together to form one group, which was named picture. Next, the Kruskal–Wallis test (*p*-value: 0.05) testing the picture condition against the text condition was applied separately to each feature. The total means for both conditions and each feature, together with the *p*-value levels, are presented in Figure 7. In the figure, seven out of nine features (attention, anger, sadness, disgust, joy, surprise, and fear) significantly differed between the two analyzed conditions.

Five of the significant effects found in the analysis agreed with our preliminarily expectations and are straightforward to explain. The text-reading period required more attention than the picture-presentation period because the subjects were forced to read and understand the text in order to provide the correct answers to the questions following each section of the text. The higher disgust in the picture presentation period was also expected, since some of the negative pictures were perceived as revolting for most subjects, which contrasted the neutral tone of the text. The significant increase in the anger feature in the picture presentation period is also easy to explain. The pictures appearing in front of the subject’s eyes and obscuring the text interrupted the current cognitive task performed by the subject and were hence perceived as annoying. The higher anger was negatively correlated with the next significant feature, joy, which explains its smaller value in the picture presentation period.

The next effect that we detected was less sadness in the picture presentation period. Once again, the explanation of this effect is straightforward. Sadness is a negative emotion that reflects a person’s avoidance tendency. Anger is also a negative emotion, but it reflects the motivation to engage (approach tendency). Since a person cannot withdraw and engage at the same time, one emotion has to prevail. In the case of interrupting a person’s ongoing cognitive process, anger is usually much stronger than eventual sadness; as a result, the pop-up pictures induced more anger and less sadness.

The emotional feature that was the most unexpected to us was the feature depicting the subject’s surprise. At the beginning of the experiment, we were sure that since the picture presentation time was random, the subject should be surprised each time when a picture appeared on the screen. Our anticipation was found to be false: the subjects were significantly more surprised by the text than by the pictures. One possible explanation of this effect might be that the symptoms of surprise appeared in both the text-reading and picture presentation periods. However, during the text-reading period, the surprise emotion had chances to develop; in the picture presentation period, it was quickly overcome by anger associated with the interruption of the cognitive task.

**Figure 8:**
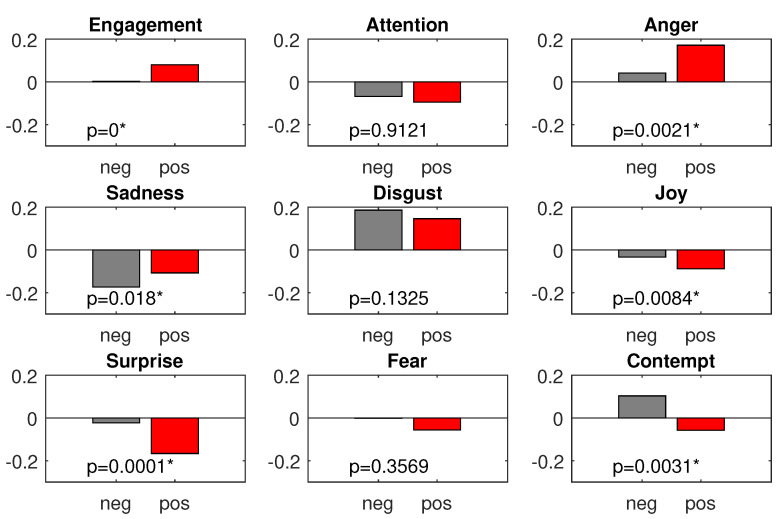
Emotional features: total means for negative and positive picture presentation conditions; the significant differences in both conditions (at 0.05 level) are denoted with an asterisk.

The last significant effect that we identified during the analysis was the higher fear in the text-reading period. This effect was the opposite of our expectation. The set of negative pictures used in our experiment also included pictures with fearful content. Hence, we expected those pictures to evoke fear in the subjects. However, the fearful pictures induced some fear but not enough to counteract the nonfearful positive pictures (compare Figure 8).

Figure 7 shows the differences between the text-reading and the text presentation periods. As we mentioned above, the majority of the identified effects were as expected. However, the main aim of our analysis was not to observe what happened when we presented a picture on the screen but to determined whether the valence of the picture could induce differences in the emotions felt by the subjects. To this end, we performed another set of Kruskal–Wallis tests (*p*-value: 0.05), one test per feature. This time, we tested all periods when the positive pictures were presented (positive condition) against all periods when the negative pictures were presented (negative condition). The total means for both conditions and each feature, together with the *p*-value levels, are presented in Figure 8.

From the figure, six out of nine features (engagement, anger, sadness, joy, surprise, and contempt) significantly differed between the two analyzed conditions. The comparison of the grand means obtained for three of those features showed the assumed tendency: the negative pictures were perceived as more surprising, more contemptuous, and sadder than positive ones.

Three other significant effects, higher anger and engagement and lower joy for positive pictures, were the opposite of expected. At the beginning of the experiment, we assumed that positively loaded content would be able to attenuate the irritation induced by interrupting the user’s main task: the text-reading task. Hence, we assumed that anger would be significantly higher for negative than for positive pictures. However, the more anger in the positive picture presentation period was one of the most vivid effects in the experiment. Since engagement and joy are highly correlated with anger (engagement presented positive and joy presented negative correlation), higher anger during the positive picture presentation period also directly explains the two remaining effects: higher engagement and lower joy.

### 3.3. Discussion

Despite marketing activity being associated with digital environments and commercial aspects being important for portals, games, and social platforms, user experience makes it important to keep those environments as user-friendly as possible. The types of content and techniques used have an impact on the obtained effects as well as on user perception and attitudes toward brands. Earlier attempts showed that the level of intrusiveness could be reduced by adding emotional appeal to video ads [14]. Earlier results based on subjective assessments from user surveys showed that positive emotional content generated a more positive attitude toward the advertising content and the brand. To avoid subjectivity, we based our study on objective measures derived from eye tracking and facial emotion recognition. With those measures, we obtained effects contradictory to previously reported findings: simple positive content based on, e.g., animals, cats, or nice views, has no ability to reduce the irritation caused by disruptive content.

For the reduction in the negative impact due to disruptive content, the intensity of stimuli and the level of attention induced during interruptions observed for negative content were more effective than positive emotional load. This supports earlier results [1] showing that if information as well as an ad are regarded as important, interesting, or intriguing for the viewer, they feel a lower irritation level due to the interruption. The content with potentially negative appeal from different categories, such as dangerous animals, disgusting pictures, or sad scenes, generated higher attention as measured with eye tracking. This results in capturing information with focal vision, which is important for effective communication [65]. This is important from the perspective of practical applications because attention is one of key factors recognized as important for brand memory [66] and attitudes [67].

Hence, one of the conclusions of our experiment is that user attention was dependent on the valence of the emotional content. In the case of positive pictures, the attention was more ambient than for the negative ones, which were characterized with more focal attention. This conclusion is supported by the fixation time. The long fixation time corresponded to the pictures with a negative load, opposite to the short fixation time observed for the positive pictures. The analysis of saccadic eye movements (saccades velocity) provided more insight into the users’ cognitive states and emotions: it showed greater cognitive load and irritation (which is consistent with the results in [37]); surprisingly, this was not found in the case of negative pictures but in the case of positive ones. These results might be a consequence of the pictures being so intriguing and surprising that they eliminated the irritation resulting from the interruption of the cognitive process, as opposed to positive pictures. The conclusions formulated on the basis of eye patterns confirm the facial features analysis. Here, irritation, measured by anger level, was much higher when pictures with positive valence were presented.

These results are consistent with those of earlier studies [68], emphasizing that negative stimuli are treated as emotionally more intensive than positive stimuli. Cognitive process interruption is intense on its own, and content with positive emotional load did not have enough intensity to replace its negative impact.

Our experiment was based on several methodological choices. Firstly, we used affective pictures from a standardized database instead of real advertisements. We decided to go in this direction, since those pictures were accompanied by an identified emotional load. Moreover, they allowed us to keep an emotional load not disturbed by marketing content, brand names, or product altitudes. On the other hand, our choice might narrow the scalability of our results, as true-marketing stimuli can have a slightly different influence on the users.

Secondly, we used an eye-tracking system and facial emotion recognition software to measure the emotional reaction of respondents. Both approaches are usually the first choice in the experiments focused on emotion recognition, although the methods that measure directly the biosignals generated by the human nervous system, such as electroencephalography, electrocardiography, electromyography, or galvanic skin response, usually provide more precise measurements. However, those methods, are characterized by higher invasiveness and smaller applicability, which makes them less practical and harder to use in outside-laboratory conditions.

Thirdly, we focused only on one cognitive task, a text-reading task that is typical for portals or blogs being the main target for marketing activity. This choice might influence the transition of our results to non-text content that provides different levels of user engagement. Moreover, we used neutral texts, while texts within websites might have a positive or negative emotional load. Consistency or inconsistency between text and content used during interruption of user cognitive process can also have an impact on differences in users’ reactions.

One possible limitation of the results presented in this paper is related to the scaling intensity of both positive and negative content. We used two classes of images, and in both classes, different levels of positive or negative impact could be distinguished. It opens additional directions for future research on the impact of content type and task difficulty on user emotions.

## 4. Conclusions

The study whose results are presented in the paper was designed to provide a better understanding of the impact of emotional content used in the process of creating marketing content on the users’ perception. To achieve this aim, we conducted the cognitive experiment with affective pictures that enabled us to study users’ visual attention and their emotional response to visual stimuli.

By studying both factors simultaneously, we were able to find a more compact and unified answer to the question on how to prepare the marketing content to reduce a user irritation and limit ad avoidance.

Our experiment clearly shows that the user attention depends on the valence of the emotional content. In the case of positive pictures, the attention was more ambient than for the negative ones, which were characterized by more focal attention. This conclusion was also supported by the fixation time, which was higher for negative pictures. The conclusions formulated on the basis of eye patterns were confirmed also by the facial features analysis. Here, irritation, measured by anger level, was much higher when pictures with positive valence were presented.

The presented results show the possibility of replacing emotions induced by interruptions using content with stronger emotional load such as shocking or scary pictures. This creates implications for practice when adjusting the emotional intensity of the content used for the marketing technique and intensity of other stimuli within the user’s environment. We do not suggest using shocking content in typical campaigns; instead, we propose searching for methods of attracting user attention by emotionally intensifying the disruptive content, because this might lower user irritation.

## References

[1] Edwards S.M., Li H., Lee J.H. (2002). Forced exposure and psychological reactance: Antecedents and consequences of the perceived intrusiveness of pop-up ads. J. Advert..

[2] Goodrich K., Schiller S.Z., Galletta D. (2015). Consumer reactions to intrusiveness of online-video advertisements: Do length, informativeness, and humor help (or hinder) marketing outcomes?. J. Advert. Res..

[3] Li H., Edwards S.M., Lee J.H. (2002). Measuring the intrusiveness of advertisements: Scale development and validation. J. Advert..

[4] Krugman H.E. (1983). Television program interest and commercial interruption. J. Advert. Res..

[5] Hussain D., Lasage H. (2014). Online video advertisement avoidance: Can interactivity help?. J. Appl. Bus. Res. (JABR).

[6] Ferreira C., Michaelidou N., Moraes C., McGrath M. (2017). Social media advertising: Factors influencing consumer ad avoidance. J. Cust. Behav..

[7] Bang H., Kim J., Choi D. (2018). Exploring the effects of ad-task relevance and ad salience on ad avoidance: The moderating role of internet use motivation. Comput. Hum. Behav..

[8] Kim N.Y., Sundar S.S. (2010). Relevance to the rescue: Can “smart ads” reduce negative response to online ad clutter?. J. Mass Commun. Q..

[9] Teixeira T., Picard R., El Kaliouby R. (2014). Why, when, and how much to entertain consumers in advertisements? A web-based facial tracking field study. Mark. Sci..

[10] Van Doorn J., Hoekstra J.C. (2013). Customization of online advertising: The role of intrusiveness. Mark. Lett..

[11] Mishra S., Malhotra G. (2020). The gamification of in-game advertising: Examining the role of psychological ownership and advertisement intrusiveness. Int. J. Inf. Manag..

[12] Kim S. (2015). Effects of Ad-Video Similarity, Ad Location, and User Control Option on Ad Avoidance and Advertiser-Intended Outcomes of Online Video Ads. Ph.D. Thesis.

[13] Campbell C., Mattison Thompson F., Grimm P.E., Robson K. (2017). Understanding why consumers don’t skip pre-roll video ads. J. Advert..

[14] Hegner S.M., Kusse D.C., Pruyn A.T. (2016). Watch it! The influence of forced pre-roll video ads on consumer perceptions. Advances in Advertising Research (Vol. VI).

[15] Öhman A., Flykt A., Esteves F. (2001). Emotion drives attention: Detecting the snake in the grass. J. Exp. Psychol. Gen..

[16] Buodo G., Sarlo M., Palomba D. (2002). Attentional resources measured by reaction times highlight differences within pleasant and unpleasant, high arousing stimuli. Motiv. Emot..

[17] Schimmack U., Derryberry D.E. (2005). Attentional interference effects of emotional pictures: Threat, negativity, or arousal?. Emotion.

[18] Fenske M.J., Eastwood J.D. (2003). Modulation of focused attention by faces expressing emotion: Evidence from flanker tasks. Emotion.

[19] Fox E., Russo R., Georgiou G.A. (2005). Anxiety modulates the degree of attentive resources required to process emotional faces. Cogn. Affect. Behav. Neurosci..

[20] Derryberry D., Tucker D.M. (1994). Motivating the Focus of Attention. https://psycnet.apa.org/record/1994-97332-007.

[21] Wadlinger H.A., Isaacowitz D.M. (2006). Positive mood broadens visual attention to positive stimuli. Motiv. Emot..

[22] Velichkovsky B.M., Joos M., Helmert J.R., Pannasch S. (2005). Two visual systems and their eye movements: Evidence from static and dynamic scene perception. Proceedings of the XXVII Conference of the Cognitive Science Society.

[23] Krejtz K., Çöltekin A., Duchowski A., Niedzielska A. (2017). Using Coefficient to Distinguish Ambient/Focal Visual Attention during Cartographic Tasks. J. Eye Mov. Res..

[24] Vogt J., De Houwer J., Koster E.H., Van Damme S., Crombez G. (2008). Allocation of spatial attention to emotional stimuli depends upon arousal and not valence. Emotion.

[25] Lang P.J., Bradley M.M., Cuthbert B.N. (2008). International Affective Picture System (IAPS): Affective Ratings of Pictures and Instruction Manual.

[26] Britton J.C., Taylor S.F., Sudheimer K.D., Liberzon I. (2006). Facial expressions and complex IAPS pictures: Common and differential networks. Neuroimage.

[27] Bradley M.M., Lang P.J. (1994). Measuring emotion: The self-assessment manikin and the semantic differential. J. Behav. Ther. Exp. Psychiatry.

[28] Marandi R.Z., Sabzpoushan S.H. (2015). Qualitative modeling of the decision-making process using electrooculography. Behav. Res. Methods.

[29] Hopstaken J.F., van der Linden D., Bakker A.B., Kompier M.A., Leung Y.K. (2016). Shifts in attention during mental fatigue: Evidence from subjective, behavioral, physiological, and eye-tracking data. J. Exp. Psychol. Hum. Percept. Perform..

[30] Marandi R.Z., Sabzpoushan S.H. (2014). Using eye movement analysis to study auditory effects on visual memory recall. Basic Clin. Neurosci..

[31] Keller G.B., Bonhoeffer T., Hübener M. (2012). Sensorimotor mismatch signals in primary visual cortex of the behaving mouse. Neuron.

[32] Helo A., Rämä P., Pannasch S., Meary D. (2016). Eye movement patterns and visual attention during scene viewing in 3-to 12-month-olds. Vis. Neurosci..

[33] Di Stasi L.L., McCamy M.B., Catena A., Macknik S.L., Canas J.J., Martinez-Conde S. (2013). Microsaccade and drift dynamics reflect mental fatigue. Eur. J. Neurosci..

[34] Stern J.A., Boyer D., Schroeder D. (1994). Blink rate: A possible measure of fatigue. Hum. Factors.

[35] Marandi R.Z., Madeleine P., Omland Ø., Vuillerme N., Samani A. (2018). Reliability of oculometrics during a mentally demanding task in young and old adults. IEEE Access.

[36] Marandi R.Z., Madeleine P., Omland Ø., Vuillerme N., Samani A. (2018). Eye movement characteristics reflected fatigue development in both young and elderly individuals. Sci. Rep..

[37] Behroozi M., Lui A., Moore I., Ford D., Parnin C. Dazed: Measuring the cognitive load of solving technical interview problems at the whiteboard. Proceedings of the 40th International Conference on Software Engineering: New Ideas and Emerging Results.

[38] Series B. (2002). Methodology for the Subjective Assessment of the Quality of Television Pictures. https://www.itu.int/rec/R-REC-BT.500-11-200206-S.

[39] Alarcao S.M., Fonseca M.J. (2017). Emotions recognition using EEG signals: A survey. IEEE Trans. Affect. Comput..

[40] Das P., Khasnobish A., Tibarewala D. Emotion recognition employing ECG and GSR signals as markers of ANS. Proceedings of the 2016 Conference on Advances in Signal Processing (CASP).

[41] Jerritta S., Murugappan M., Wan K., Yaacob S. (2014). Emotion recognition from facial EMG signals using higher order statistics and principal component analysis. J. Chin. Inst. Eng..

[42] Kim J., André E. (2008). Emotion recognition based on physiological changes in music listening. IEEE Trans. Pattern Anal. Mach. Intell..

[43] Wu G., Liu G., Hao M. The analysis of emotion recognition from GSR based on PSO. Proceedings of the 2010 International Symposium on Intelligence Information Processing and Trusted Computing.

[44] Lim J.Z., Mountstephens J., Teo J. (2020). Emotion recognition using eye-tracking: Taxonomy, review and current challenges. Sensors.

[45] Kleinsmith A., Bianchi-Berthouze N. (2007). Recognizing affective dimensions from body posture. International Conference on Affective Computing and Intelligent Interaction.

[46] El Ayadi M., Kamel M.S., Karray F. (2011). Survey on speech emotion recognition: Features, classification schemes, and databases. Pattern Recognit..

[47] Clark E.A., Kessinger J., Duncan S.E., Bell M.A., Lahne J., Gallagher D.L., O’keefe S.F. (2020). The Facial Action Coding System for Characterization of Human Affective Response to Consumer Product-Based Stimuli: A Systematic Review. Front. Psychol..

[48] Wegrzyn M., Vogt M., Kireclioglu B., Schneider J., Kissler J. (2017). Mapping the emotional face. How individual face parts contribute to successful emotion recognition. PLoS ONE.

[49] Lewinski P., den Uyl T.M., Butler C. (2014). Automated facial coding: Validation of basic emotions and FACS AUs in FaceReader. J. Neurosci. Psychol. Econ..

[50] Lien J.J., Kanade T., Cohn J.F., Li C.C. Automated facial expression recognition based on FACS action units. Proceedings of the Third IEEE International Conference on Automatic Face and Gesture Recognition.

[51] Skiendziel T., Rösch A.G., Schultheiss O.C. (2019). Assessing the convergent validity between the automated emotion recognition software Noldus FaceReader 7 and Facial Action Coding System Scoring. PLoS ONE.

[52] Cohn J.F., Ambadar Z., Ekman P. (2007). Observer-based measurement of facial expression with the Facial Action Coding System. Handb. Emot. Elicitation Assess..

[53] Friesen E., Ekman P. (1978). Facial action coding system: A technique for the measurement of facial movement. Palo Alto.

[54] Hjortsjö C.H. (1969). Man’s Face and Mimic Language.

[55] Ekman P., Friesen W., Hager J. (2002). Facial Action Coding System: The Manual on CD-ROM. Instructor’s Guide.

[56] Ekman P., Rosenberg E., Hager J. (1998). Facial Action Coding System Interpretive Database (FACSAID).

[57] Li J., Oussalah M. Automatic face emotion recognition system. Proceedings of the 2010 IEEE 9th International Conference on Cyberntic Intelligent Systems.

[58] Zhang H., Jolfaei A., Alazab M. (2019). A face emotion recognition method using convolutional neural network and image edge computing. IEEE Access.

[59] Zhong Y., Sun L., Ge C., Fan H. (2021). HOG-ESRs Face Emotion Recognition Algorithm Based on HOG Feature and ESRs Method. Symmetry.

[60] Küntzler T., Höfling T.T.A., Alpers G.W. (2021). Automatic facial expression recognition in standardized and non-standardized emotional expressions. Front. Psychol..

[61] Brodny G., Kołakowska A., Landowska A., Szwoch M., Szwoch W., Wróbel M.R. Comparison of selected off-the-shelf solutions for emotion recognition based on facial expressions. Proceedings of the 2016 9th International Conference on Human System Interactions (HSI).

[62] Stöckli S., Schulte-Mecklenbeck M., Borer S., Samson A.C. (2018). Facial expression analysis with AFFDEX and FACET: A validation study. Behav. Res. Methods.

[63] Kulke L., Feyerabend D., Schacht A. (2020). A comparison of the Affectiva iMotions Facial Expression Analysis Software with EMG for identifying facial expressions of emotion. Front. Psychol..

[64] Di Stasi L.L., Antolí A., Cañas J.J. (2011). Main sequence: An index for detecting mental workload variation in complex tasks. Appl. Ergon..

[65] Drèze X., Hussherr F.X. (2003). Internet advertising: Is anybody watching?. J. Interact. Mark..

[66] Guitart I.A., Hervet G., Hildebrand D. (2019). Using eye-tracking to understand the impact of multitasking on memory for banner ads: The role of attention to the ad. Int. J. Advert..

[67] Chatterjee P. (2008). Are Unclicked Ads Wasted? Enduring Effects of Banner and Pop-Up Ad Exposures on Brand Memory and Attitudes. J. Electron. Commer. Res..

[68] Vaish A., Grossmann T., Woodward A. (2008). Not all emotions are created equal: The negativity bias in social-emotional development. Psychol. Bull..

